# Right Trochlear Nerve Palsy as an Uncommon Manifestation of Arachnoid Cyst

**DOI:** 10.7759/cureus.33579

**Published:** 2023-01-10

**Authors:** Ruknesvary Subramaniam, Wan Hazabbah Wan Hitam, Khairy Shamel Sonny Teo, Chandran Nadarajan

**Affiliations:** 1 Department of Ophthalmology and Visual Science, School of Medical Sciences, Health Campus, Universiti Sains Malaysia, Kubang Kerian, MYS; 2 Department of Radiology, School of Medical Sciences, Health Campus, Universiti Sains Malaysia, Kubang Kerian, MYS

**Keywords:** intraneural cyst, 4th cranial nerve palsy, diplopia, superior oblique muscle palsy, arachnoid cyst

## Abstract

The majority of arachnoid cysts are congenital intracranial lesions that develop in the early embryonic stages as a result of a slight irregularity in the cerebrospinal fluid's (CSF) passage through the embryonic mesenchyme. Most of the time, these cysts are asymptomatic all throughout life. Diplopia caused by an arachnoid cyst is extremely rare. We present a rare event of isolated fourth nerve palsy in a 56-year-old woman brought on by an intracranial arachnoid cyst. Her only presenting symptom was vertical diplopia for one week. She denied any history of trauma. Ocular motility revealed limitation of abduction in her right eye. We proceeded with neuroimaging and the magnetic resonance imaging (MRI) confirmed the presence of a well-circumscribed left retro-cerebellar lesion which follows the CSF signal intensity in all sequences causing compression onto the posterior aspect of the left cerebellum, keeping with the diagnosis of an arachnoid cyst. This uncommon pathology tends to be difficult to diagnose and treat.

## Introduction

Arachnoid cysts are fluid-filled breaks in the arachnoid layer with contents similar to the cerebrospinal fluid (CSF). It is not a neurodegenerative disorder but more of an underlying defect of the constitutes of the arachnoid layer which is most often congenital in nature. They are usually associated with other illnesses but can also occur sporadically in some cases [1]. Based on a few previous studies, arachnoid cysts were first reported about 200 years ago and the prevalence of arachnoid cysts currently is between 1.4 and 1.7% in adults and about 2.6% in children [2,3]. The occurrence of arachnoid cysts is higher among females. There are two different ways to classify arachnoid cysts which are either primary or secondary cysts. Development of anomalous collections of CSF as a result of the splitting of arachnoid membranes in utero gives rise to primary cysts whereas secondary cysts are less commonly occurring and usually arise after surgery, trauma, infection, or intracranial hemorrhage [4]. Arachnoid cysts normally occur in the posterior fossa and middle cerebral fossa, especially in the Sylvian fissure, and are typically asymptomatic throughout life [5,6]. This case report was done with the objective of reporting a rare ocular manifestation of an arachnoid cyst in an adult.

This case was presented as a poster at the 4th USIM International Health E-conference 2020 (IHEC 2020) from December 16-17, 2020.

## Case presentation

A 56-year-old woman who had underlying hypertension presented to us complaining of vertical diplopia for a week. Her symptom began suddenly at rest and persisted. There had been no recent trauma or illness. She denied any reduced vision, floaters, or metamorphopsia. She denied having a headache, seizure, nausea, or persistent vomiting. Her medical and family histories were non-contributory.

Aside from the ocular findings, her general and neurologic examination results were normal. A neuro-ophthalmology test found that both eyes' visual acuity was 6/6. Ocular motility in the right eye exhibited very little abduction restriction. Except when she is looking up, she had diplopia in all of her gazes. Her field of vision was normal. The anterior segment had no notable features. Examination of both eye fundus disclosed no papilledema and was insignificant. No other cranial nerve weaknesses were noted. The presence of right superior oblique paresis was confirmed by the Hess test.

**Figure 1:**
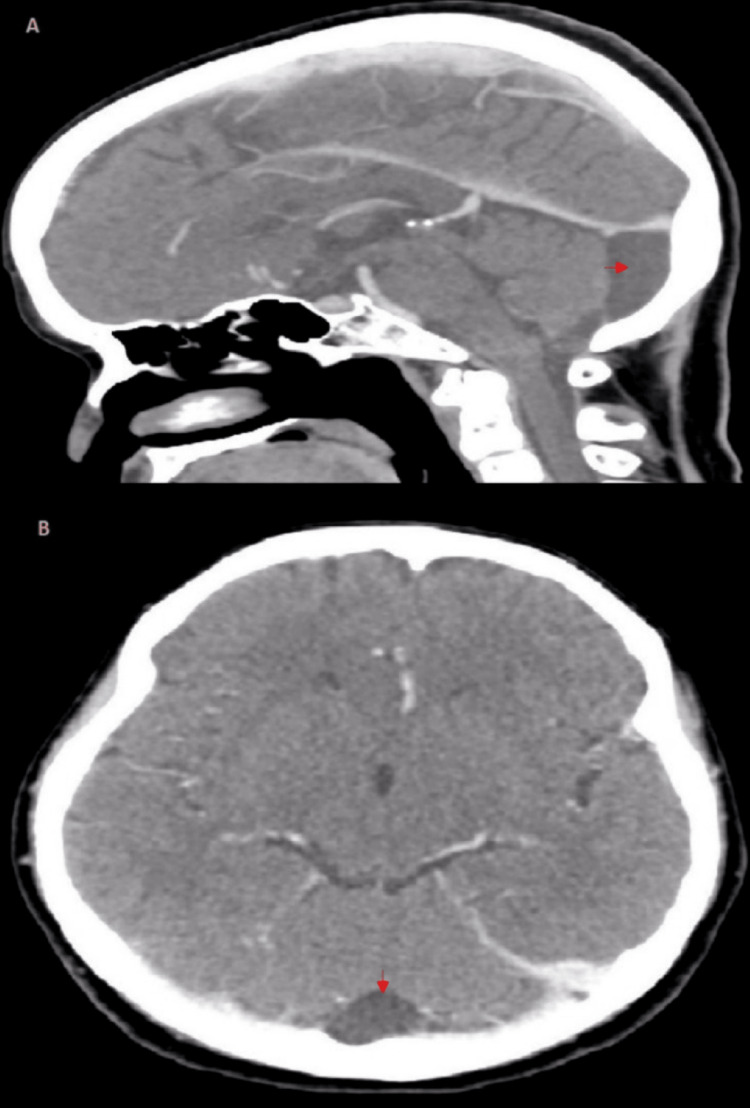
Sagittal (A) and axial (B) view of the contrasted CT images of the brain shows fluid density lesion in the posterior fossa region (red arrow).

**Figure 2:**
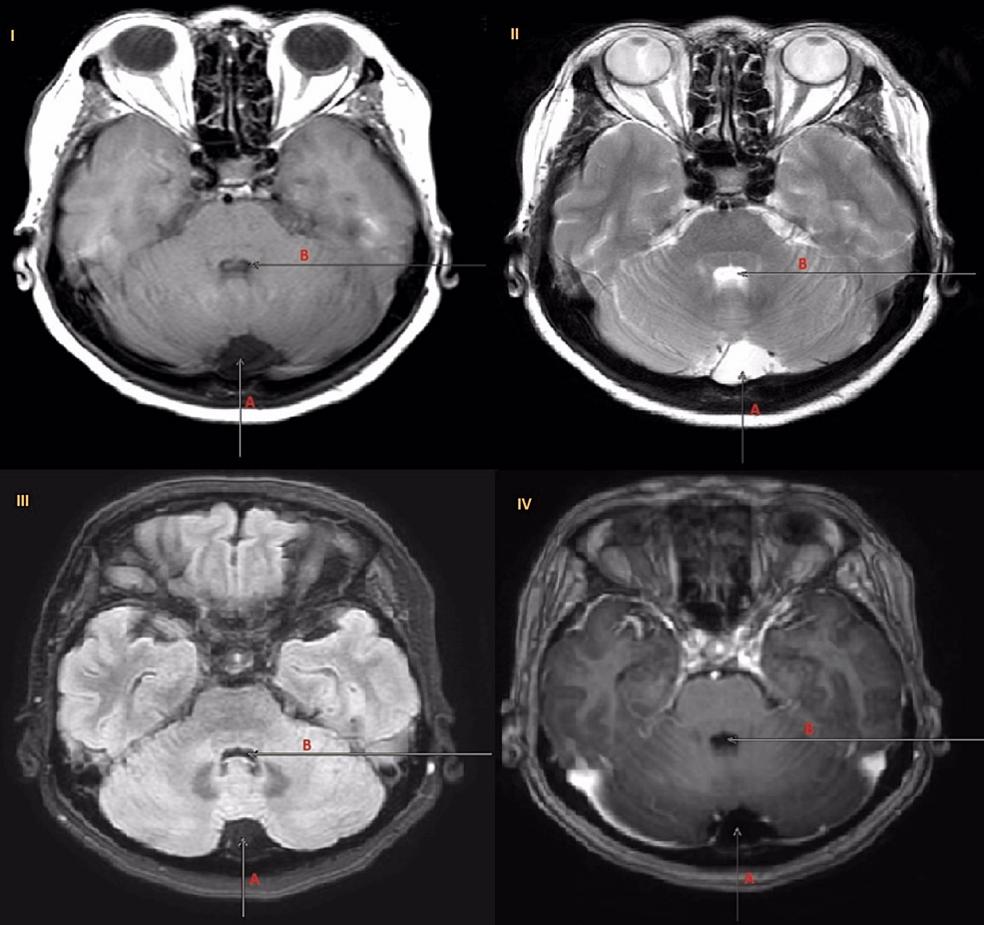
A cystic lesion in the posterior fossa (arrow A) with similar signal intensity as the CSF in the fourth ventricle (arrow B) shown in MRI in different views. I: T1-weighted image, II: T2-weighted image, III: Flair sequence image, IV: Post-gadolinium image

We proceeded with neuroimaging to identify the lesion causing the trochlear nerve palsy. A well-defined non-enhancing hypodense lesion at the retro-cerebellar area was seen on the Computed Tomography (CT) scan (Figures 1A, 1B), which strongly suggests an arachnoid cyst. It creates a minor mass effect on neighboring cerebellar folia. After being evaluated by our neurosurgical team, she was booked for an outpatient magnetic resonance imaging (MRI) to confirm the diagnosis as there were no other neurological abnormalities. The MRI showed the presence of a well-circumscribed left retro-cerebellar lesion which follows the CSF signal intensity in all sequences (Figure 2). There is also mild compression on the posterior aspect of the left cerebellum. There is no focal enhancing brain or orbit lesion. During her subsequent follow-ups, her diplopia was improving to almost non-existent at times, and there was no neurological deficit. As such, no immediate surgical intervention was planned.

## Discussion

Arachnoid cysts account for approximately 1% of all intracranial mass lesions [7]. An isolated cranial nerve palsy caused by an intraneural arachnoid cyst is extremely unusual. While 75% of symptomatic cysts are diagnosed in children, asymptomatic cysts are more frequently found incidentally in adults [8]. According to a study by Ohtsuka, arachnoid cysts are usually located in the posterior fossa or in the middle cranial fossa which most often involves the Sylvian fissure, and the study also highlighted that diplopia caused by arachnoid cyst is indeed very rare [9]. Studies have also revealed that acquired unilateral or bilateral superior oblique palsy is most frequently brought on by trauma. This makes this case report unique as the patient did not suffer any trauma and her only chief complaint was diplopia.

The preferred diagnostic method for assessing these lesions is MRI, using axial, coronal, and sagittal sections, as detailed above [10]. CSF flow dynamic study in MRI of our patient revealed the presence of flow within the retro cerebellar cyst, representing communication within the subarachnoid space. There is also compression on the posterior aspect of the left cerebellum. Because the trochlear nerve (cranial nerve IV) is the only motor cranial nerve that exits from the dorsal side of the brainstem, compression on the cerebellum could lead to compression on the posterior aspect of the brainstem, resulting in cranial nerve IV palsy. According to a study done in 2022, most arachnoid cysts do not need to be treated. However, when significant neurological symptoms manifest, most neurosurgeons recommend surgery. The approach is determined by the anatomic location and the patient's condition [11]. The surgical treatment of arachnoid cysts is an ongoing debate. The surgeon is faced with several surgical options: Drainage via transcranial puncture or trephination, cyst-peritoneal shunting, craniotomy for cyst excision or marsupialization, and hydrocephalus treatment with ventricular-peritoneal shunting while preserving the cyst [10]. In our patient, neurosurgical intervention was not advocated given the stable size of the cyst and the absence of any additional neurological deficits.

## Conclusions

In conclusion, we provide a unique instance of an arachnoid cyst presenting as acquired isolated fourth nerve palsy in an adult. The case highlights the diagnostic value of neuroimaging when a patient presents with such subtle symptoms. To the best of our knowledge, this is the first account of an adult in Southeast Asia with an arachnoid cyst triggering fourth nerve palsy, with only about ten other reports in total worldwide.
